# Weight-based nutritional diagnosis of Mexican children and adolescents with neuromotor disabilities

**DOI:** 10.1186/1756-0500-5-218

**Published:** 2012-07-04

**Authors:** Rodrigo Vega-Sanchez, Maria de la Luz Gomez-Aguilar, Karime Haua, Guadalupe Rozada

**Affiliations:** 1 Department of Nutrition Research, Instituto Nacional de Perinatología Isidro Espinosa de los Reyes, Mexico City, Mexico; 2 Nutrition Department, Centro de Rehabilitación Infantil Teletón, Estado de Mexico, Mexico; 3 Independent consultant, Mexico City, Mexico

**Keywords:** Nutrition, Obesity, Undernutrition, Children, Adolescents, Disabilities

## Abstract

**Background:**

Nutrition related problems are increasing worldwide but they have scarcely been evaluated in people with neuromotor disabilities, particularly in developing countries. In this study our aim was to describe the weight-based nutritional diagnoses of children and adolescents with neuromotor disabilities who attended a private rehabilitation center in Mexico City.

**Methods:**

Data from the first visit’s clinical records of 410 patients who attended the Nutrition department at the Teleton Center for Children Rehabilitation, between 1999 and 2008, were analyzed. Sex, age, weight and height, length or segmental length data were collected and used to obtain the nutritional diagnosis based on international growth charts, as well as disability-specific charts. Weight for height was considered the main indicator.

**Results:**

Cerebral palsy was the most frequent diagnosis, followed by spina bifida, muscular dystrophy, and Down’s syndrome. Children with cerebral palsy showed a higher risk of presenting low weight/undernutrition (LW/UN) than children with other disabilities, which was three times higher in females. In contrast, children with spina bifida, particularly males, were more likely to be overweight/obese (OW/OB), especially after the age of 6 and even more after 11. Patients with muscular dystrophy showed a significantly lower risk of LW/UN than patients with other disabilities. In patients with Down’s syndrome neither LW/UN nor OW/OB were different between age and sex.

**Conclusions:**

This is the first study that provides evidence of the nutritional situation of children and adolescents with neuromotor disabilities in Mexico, based on their weight status. Low weight and obesity affect a large number of these patients due to their disability, age and sex. Early nutritional diagnosis must be considered an essential component in the treatment of these patients to prevent obesity and malnutrition, and improve their quality of life.

## Background

Nutrition related problems, such as obesity and undernutrition, have greatly increased worldwide over the last few decades, and represent a major cause of morbidity and mortality, not only in adults but among children and adolescents as well. In Mexico, 26% of children between ages 5 and 11 show some degree of overweight or obesity, and around 10% are undernourished [1].

Despite extensive research in this area, studies evaluating nutrition related problems in people with neuromotor disabilities have mainly been done in developed countries [2-7] and thus data about this population group is scarce in developing countries. For Latin America the only data available come from Brazil and Chile, where disabled children (particularly those with cerebral palsy) tend to be severely undernourished, contrasting with other countries where obesity is more prevalent [7-9].

People with neuromotor disabilities (defined as sequels of a condition that affects the central or peripheral nervous system or both, and also the skeletal muscle system [10]) account for more than one million individuals in Mexico [11] of whom no nutritional status data is available. In this study, our aim was to analyze the nutritional status of a group of Mexican children and adolescents with neuromotor disabilities, as inferred by their weight status.

## Results

Sex frequencies and age ranges for the different kinds of disabilities are shown in Table 1. Sex distribution was homogeneous with a median age of 7.09 years ranging from 4 months to 17.5 years.

**Table 1:** Sex and age characteristics within most frequent disability diagnoses of patients attending the Nutrition department of CRIT for the first time

Diagnosis (ICD-10 code)	Total cases	Median age	Male	Female
	(% of all cases)	in years	(% within group)	(% within group)
	n = 410	(min-max)	n = 236	n = 174
Cerebral palsy (G80)	210 (51.2)	7.9 (0.9-17.3)	119 (50.4)	91 (49.6)
Spastic quadriplegic cerebral palsy (G80.0)	132 (32.2)	8.2 (1.1-17.2)	78 (59.1)	54 (40.9)
Cerebral palsy, unspecified (G80.9)	35 (8.5)	8.3 (0.9-15.8)	17 (48.6)	18 (51.4)
Other cerebral palsy (mixed) (G80.8)	27 (6.6)	5.7 (3.0-15.7)	17 (63.0)	10 (37.0)
Ataxic cerebral palsy (G80.4)	9 (2.2)	9.9 (2.8-16.8)	4 (44.4)	5 (55.6)
Dyskinetic cerebral palsy (G80.3)	7 (1.7)	8.2 (1.6-17.3)	3 (42.9)	4 (57.1)
Spina bifida, unspecified [myelomeningocele] (Q05.9)	66 (16.1)	4.5 (1.2-17.5)	37 (56.0)	29 (44.0)
Muscular dystrophy (G71.0)	24 (5.9)	9.0 (1.9-17.2)	19 (79.2)**	5 (20.8)**
Down’s syndrome, unspecified (Q90.9)	21 (5.1)	4.1 (0.3-12.7)	13 (61.9)	8 (38.1)
Spastic tetraplegia (G82.4)	11 (2.7)	8.3 (2.0-14.4)	7 (63.6)	4 (36.4)
Arthrogryposis multiplex congenita (Q74.3)	6 (1.5)	8.0 (5.2-16.1)	4 (66.7)	2 (33.3)
Unspecified mental retardation (F79)	6 (1.5)	7.8 (4.3-10.9)	2 (33.3)	4 (66.7)
Other generalized epilepsy and epileptic syndromes (Lennox-Gastaut syndrome) (G40.4)	5 (1.2)	4.2 (1.1-11.8)	3 (60.0)	2 (40.0)
Disorder of brain, unspecified (G93.9)	5 (1.2)	4.6 (2.1-13.1)	1 (20.0)	4 (80.0)
Hydrocephalus, unspecified (G91.9)	4 (1.0)	2.2 (0.6-7.5)	4 (100.0)	0
Microcephaly (Q02)	4 (1.0)	5.0 (1.8-6.8)	0	4 (100.0)
Total cases	410		236	174

Pearson’s Chi squared test was used to compare frequencies of males and females within the each diagnosis. The only significant difference (** p = 0.02) was found in muscular dystrophy.

Cerebral palsy, with its diverse classifications, was the most frequent diagnosis affecting half of the patients (210 cases). Within this condition, almost two thirds corresponded to spastic cerebral palsy. Unfortunately, no data was available regarding motor impairment severity, so patients could not be sorted according to the gross motor function classification system (GMFCS) [12].

The next more frequent conditions were spina bifida, muscular dystrophy and Down’s syndrome. The frequency of each of the other diagnoses represented less than 5% of the analyzed cases (Table 1).

When comparing the number of male and female patients within each disability diagnosis, muscular dystrophy showed the only significant difference, being more frequent in males than in females (19 versus 5 cases respectively, p = 0.02). This finding is expected considering that both Duchenne type (present in 10 out of 24 patients with muscular dystrophy) as well as Becker type muscular dystrophy (2 out of 24 patients) are genetic recessive disorders associated to the X chromosome and therefore affect mainly males.

**Figure 1:**
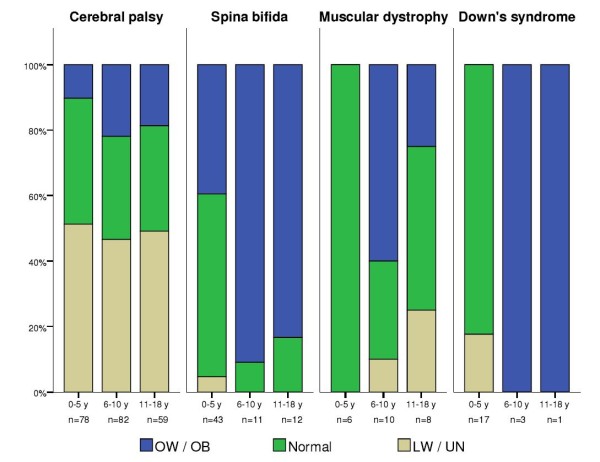
**Weight-based nutritional status of patients with the most frequent disability diagnoses by age group (in years).** The “cerebral palsy” group includes the spastic, mixed, ataxic, dyskinetic and unspecified types. LW, low weight; UN, undernutrition risk; OW, overweight; OB, obesity.

**Figure 2:**
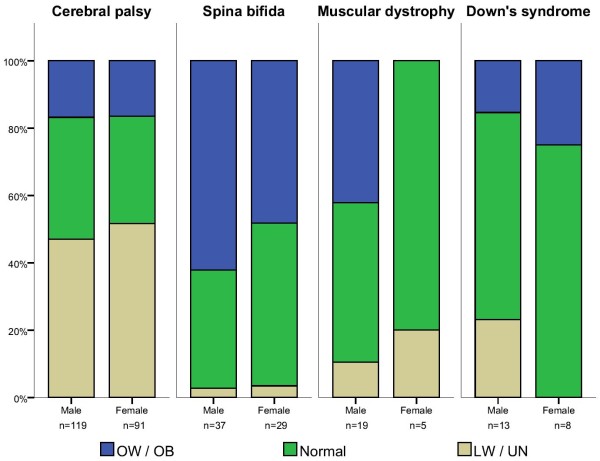
**Weight-based nutritional status of patients with the most frequent disability diagnoses by sex group.** The “cerebral palsy” group includes the spastic, mixed, ataxic, dyskinetic and unspecified types. LW, low weight; UN, undernutrition risk; OW, overweight; OB, obesity.

We then analyzed whether low weight/undernutrition risk (LW/UN) or overweight/obesity (OW/OB) were associated with specific disabilities, either by age or sex. As explained in the Methods section, this analysis was performed only for the most frequent conditions: cerebral palsy (CP), spina bifida (SB), muscular dystrophy (MD) and Down’s syndrome (DS) (Table 2). It showed clear differences among the various disabilities, as well as between age (Figure 1) and sex categories (Figure 2).

**Table 2:** Weight-based nutritional diagnosis of patients attending the Nutrition Department of CRIT for the first time by disability diagnosis and age

Diagnosis (ICD-10 code)	LW / MN		Normal		OW / OB		Total
	Male	Female	Male	Female	Male	Female	
Cerebral palsy (G80)							
0-5 years	23 (51.1)	17 (51.5)	17 (37.8)	13 (39.4)	5 (11.1)	3 (9.1)	78
6-10 years	15 (36.6)	19 (59.4)	15 (36.6)	8 (25.0)	11 (26.8)	5 (15.6)	73
11-18 years	18 (54.5)	11 (42.3)	11 (33.3)	8 (30.8)	4 (12.1)	7 (26.9)	59
Total	56	47	43	29	20	15	210
Spina bifida, unspecified [myelomeningocele] (Q05.9)							
0-5 years	1 (4.0)	1 (5.6)	12 (48.0)	12 (66.7)	12 (48.0)	5 (27.8)	43
6-10 years	0	0	0	1 (16.7)	5 (100)	5 (83.3)	11
11-18 years	0	0	1 (14.3)	1 (20.0)	6 (85.7)	4 (80.0)	12
Total	1	1	13	14	23	14	66
Muscular dystrophy (G71.0)							
0-5 years	0	0	2 (100)	4 (100)	0	0	6
6-10 years	0	1 (100)	3 (33.3)	0	6 (66.7)	0	10
11-18 years	2 (25.0)	0	4 (50.0)	0	2 (25.0)	0	8
Total	2	1	9	4	8	0	24
Down’s syndrome, unspecified (Q90.9)							
0-5 years	3 (27.3)	0	8 (72.7)	6 (100)	0	0	17
6-10 years	0	0	0	0	2 (100)	1 (100)	3
11-18 years	0	0	0	0	0	1 (100)	1
Total	3	0	8	6	2	2	21
Total of disabilities	62	49	73	53	53	31	321

Data show number of cases and parentheses show percentages within each Sex/Age category.

When grouping patients with CP, regardless of the type, we found that LW/UN was the most frequent diagnosis, accounting for 49% of the cases, while 16% of the patients with CP presented OW/OB.

Children with CP showed a higher risk of presenting LW/UN when compared to children with other disabilities. It was significantly higher in children under the age of 6 than in older children (OR = 7.57, 95% CI 2.85-20.07 and OR = 6.97, 95% CI 3.10-16.66 respectively, p < 0.01). Moreover, risk of LW/UN was three times higher in females than in males (OR = 15.31, 95% CI 4.42-52.93 and OR = 5.25, 95% CI 2.52-10.93 respectively, p < 0.01).

Similarly, children with CP who were younger than 6 showed a lower risk of presenting OW/OB (OR = 0.19, 95% CI 0.10-0.36), and also significantly less (p < 0.001) in males (OR = 0.26, 95% CI 0.13-0.51) than in females (OR = 0.33, 95% CI 0.14-0.76).

Fifty-six percent of the patients with spina bifida (SB) showed OW/OB (Table 2). In general, children with SB were less likely to have LW/UN than children with other disabilities. This was observed in children under 5 years (OR = 0.08, 95% CI 0.02-0.39) as well as in those older than 6 (OR = 0.54, 95% CI 0.47-0.62). In contrast, age was significantly associated with the risk of OW/OB. Children with SB were more likely to be overweight/obese after the age of 6 (OR = 13.32, 95% CI 5.07-34.95) when compared to those under 5 years (p < 0.001), a risk that further increased in children older than 11 (OR = 16.04, 95% CI 3.31-77.59). The risk of OW/OB from SB was significantly higher (p < 0.001) in males (OR = 7.01, 95% CI 3.23-15.20) than in females (OR = 4.66, 95% CI 1.92-11.32).

Half of the patients with MD had a normal nutritional diagnosis and one third was overweight/obese (Table 2). They showed a significantly lower risk of LW/UN than patients with other disabilities (OR = 0.24 95% CI 0.07-0.83). The small number of MD cases with LW/UN prevented from having meaningful data about its association with age or sex. OW/OB in children with MD was not associated with age (p = 0.590) or sex (p = 0.572).

In general, weight-based nutritional diagnoses of patients with Down’s syndrome (DS) showed normal distribution, with 66.7% of them classified with normal weight (Table 2). Neither LW/UN nor OW/OB were different between age groups (younger vs. older than 6, p = 0.116 / younger vs. older than 10, p = 0.990) or between males and females (p = 0.067).

Other disability diagnoses included too few cases to draw significant conclusions.

## Discussion

Nutrition related problems, such as obesity and malnutrition, represent major public health issues worldwide, both in adults and children.

Despite their importance, the prevalence of these problems in people with neuromotor disabilities, has been poorly documented, especially in developing countries such as Mexico.

In this study we describe for the first time weight-based nutritional diagnoses of Mexican children and adolescents with neuromotor disabilities, most of them between the ages of 3 and 10.

Cerebral palsy, in its various forms, was the most frequent condition in the studied population, followed by spina bifida (myelomeningocele), muscular dystrophy, Down’s syndrome, and other pathologies.

Low weight and undernutrition, as well as overweight and obesity were present in two thirds of the analyzed patients but with different frequencies between the various disabilities. In our study, patients with CP were mainly affected by low weight or undernutrition, with a prevalence of 49%. This situation is similar to that reported in Chile and Brazil where the prevalence of LW/UN in children with CP is 23.6% and 72% respectively [8,9]. When compared with reports from other non-American countries and the U.S.A., our study shows one of the highest frequencies of undernutrition in children with CP worldwide [5,6,8,9,13,14].

Female children with CP were particularly affected by this condition, with a prevalence of LW/UN three times higher than their male peers. Apart from conceivable physiological explanations, this difference could possibly result from a differential access to adequate feeding and nutrition within children’s households resembling culturally-related gender inequalities. Data from UNICEF show that children’s nutritional status is clearly related to sociocultural factors, particularly their mother’s influence in household decisions [15]. In this regard, our data raise further concerns for the nutrition of disabled girls, a vulnerable sector within a vulnerable population, making it necessary to explore the sociocultural environment of these children.

In contrast, overweight and obesity represented a greater risk for patients with spina bifida after the age of 6 and even more after 11. This observation has already been documented as a consequence of mobility limitations as well as metabolic alterations that characterize this condition, including higher body fat percentage and lower resting energy expenditure [16-18].

Although the small number of cases prevented from drawing solid conclusions, we did observe that obesity tends to increase in patients with muscular dystrophy older than 6 years old, followed by an increase in low weight and undernutrition after the age of 11. These observations correspond to the normal course of this pathology, particularly in the Duchenne type, as a result of low mobility and the loss of muscular mass respectively [19,20].

According with previous reports, we found no differences in the prevalence of obesity in children with Down’s syndrome across age groups [21-24]. However, since these patients (like those with SB) tend to develop overweight/obesity during adolescence due to several mobility, nutritional and social factors [7], their health status should be closely monitored to prevent further risks.

In the present study, disease-specific charts were used whenever possible together with the NCHS reference charts. However, physical and metabolic alterations that appear during the evolution of disabilities make it inadequate to use reference charts based on “normal” growth patterns for evaluating children with disabilities [25]. Thus it is necessary to develop disease-specific growth charts that are validated for specific populations.

Moreover, some disabilities such as CP result from a variety of particular neuromotor conditions making it difficult to group patients into specific categories. Therefore, instead of trying to adjust patients with disabilities to a population growth standard, their treatment should be tailored to the personalized growth and performance goals of each patient.

We acknowledge that our study has two main limitations. First, our population is somehow biased, since it consists of patients referred to the nutrition service of a private rehabilitation clinic, which limits the extent of our conclusions to the general disabled population. Additionally, our nutritional diagnosis is based mainly on patients’ weight, which allows only for certain inferences on their whole nutritional status and possible risk conditions.

A complete nutritional evaluation should include not only weight/height or BMI measurements but also total body fat and muscular mass determinations, either by directly measuring skin folds or using electric impedance. However, using these procedures must be carefully considered since they involve a higher cost (from specialized equipment and personnel training) and, from our experience, may be particularly invasive to patients with neuromotor disabilities.

Nevertheless, our results clearly show that, regardless of the type of neuromotor disability, nutrition related problems in these patients clearly increase with age, especially before adolescence, where the risk of presenting other metabolic complications increases [26].

Based on these results, we propose that nutritional diagnosis should constitute one of the first strategies in the rehabilitation treatment of patients with neuromotor disabilities. Additionally, providing personalized nutritional counseling and a culturally adequate nutritional education program, may allow those in charge of the daily care of these patients to identify and implement different feeding and physical activity strategies. Altogether, this could constitute an efficient way to prevent the development of nutrition related disorders and positively impact the evolution of the disability and the patient’s quality of life.

## Conclusions

Children and adolescents with neuromotor disabilities are one of the less attended populations, not only in Mexico but internationally, and their medical treatment is often focused merely on the physical rehabilitation without considering their nutritional status. This is the first report that shows the nutritional risk situation of Mexican children and adolescents with disabilities, making it evident that the prevention of undernutrition and obesity should be a priority to health service providers in order to reduce their incidence in these patients and to improve their quality of life.

## Methods

### Patient selection

All procedures involving patients were approved by the Internal Research and Ethics Committees of the Teleton Center for Children Rehabilitation in the State of Mexico (CRIT).

CRIT is located in the northwest zone of the metropolitan area of Mexico City, and is the largest private institution in Mexico that provides low cost medical and rehabilitation services to children and adolescents with neuromotor disabilities. The main criterion for the patients’ to be admitted in the CRIT is having some type of motor disability, regardless of its cause. Accordingly, all of the patients have motor disabilities even if they don’t have intellectual ones. Patients receive individualized attention and medical services according to their particular condition.

Around 27,000 patients have been seen at the CRIT since its opening in May 1999, of which approximately 15% have been referred to the Nutrition department for evaluation and diagnosis.

In this study, designed as cross-sectional, descriptive and retrospective, we analyzed the clinical records from the first visit of patients who attended the Nutrition department from June 30, 1999, to February 18, 2008. The records of around 4000 children were included, but only the first 500 cases (sorted by their internal ID number) were selected due to limited resources. From these 500 cases, only patients who attended the morning shift (between 7:00 a.m. and 3:00 p.m.) were included to avoid interpersonal variability in the anthropometric measurements. This selection process resulted in a total of 410 analyzed cases.

Sex, age, disability diagnosis, height or length, and weight data were obtained from the clinical records. Disability diagnoses were classified according to the 10th revision of the International Classification of Diseases (ICD-10, online version) [27].

### Anthropometric measurements

Weight was measured on an ADE M50610 scale (ADE, Hamburg, Germany) for patients in wheel chairs, on a Health o Meter MDC100KD-01 scale (Jarden Consumer Solutions, Boca Raton, U.S.A) for babies or infants, or on a Seca 954 chair scale (Seca, Hamburg, Germany) for the rest of the patients.

An ADE MZ10021 glass fiber metric tape (ADE, Hamburg, Germany) was used to measure height or length, while patients were lying on a bed or a stretcher. Height (or length) was measured linearly in patients whose extremities could be completely straightened, while in those patients with spasticity, contractures or deformations it was determined by segmental measures according to Stevenson [28].

All measurements were carried out by the same person and standardized according to the Habicht method in order to limit variability [29].

### Nutritional diagnosis

Since the less frequent disabilities included very few patients, nutritional diagnoses were analyzed only for the most frequent conditions (cerebral palsy, spina bifida, muscular dystrophy and Down’s syndrome), which have both a motor component as well as an intellectual one.

Nutritional diagnosis was determined using the National Center for Health Statistics (NCHS) growth charts, which are the international reference recommended by the World Health Organization. In those patients with spina bifida (myelomeningocele) or Down’s syndrome, disease-specific growth charts were also used [30,31]. Growth charts for cerebral palsy [32] were not used since they were designed for patients with quadriplegia and our study group was very heterogeneous, including patients with many kinds of motor impairments.

We used weight-for-age, weight-for-height/length, length-for-age, and body mass index (BMI)-for age charts to obtain a weight-based nutritional diagnosis. This is an indicator that does not necessarily mean a deficit in global nutritional status, although it does reflect energetic adequacies. Nevertheless, it allowed us to classify each patient’s condition as normal (adequate), low weight/undernutrition risk or overweight/obese in order to infer certain nutritional risks.

Patients between 10^th^ and 80^th^ percentiles in the various indicators were classified as normal. However, since most of the patients with neuromotor disabilities have low height or deformations in the skeletal muscle system, their height not always corresponds to that expected for their age when compared with “healthy” patients.

Therefore, if weight-for-age and/or length-for-age were below or above 10^th^ and 80^th^ percentiles, but weight-for-height/length was within that percentile range, then the nutritional diagnosis was considered normal.

Patients in the 5^th^ percentile or below were classified as low weight or undernutrition risk. Patients in the 85th percentile were considered overweight and those in the 90th percentile or above were diagnosed as obese.

### Statistical analyses

Descriptive statistics and Chi squared tests were performed to compare the sex frequencies within the disability diagnoses.

Associations between specific disabilities and nutrition diagnoses were explored with risk analyses and are shown as odds ratio (OR) and confidence intervals (CI).

Stratified analyses for low weight/undernutrition and overweight/obesity risks associated with the different disability conditions, were executed considering differences between sex and age categories using the Cochran-Mantel-Haenszel test, considering p values ≤ 0.05 as significant.

All statistical analyses were carried out using SPSS Statistics software version 17 (IBM Corporation, Somers, NY).

## Competing interests

Authors declare neither competing interests nor funding sources.

## Authors’ contributions

RVS designed study, analyzed data and wrote manuscript; MLGA evaluated patients, collected and analyzed data, and revised manuscript; KH and GR analyzed data and revised manuscript. All authors read and approved the final manuscript.
